# Portable System for Monitoring and Controlling Driver Behavior and the Use of a Mobile Phone While Driving

**DOI:** 10.3390/s19071563

**Published:** 2019-03-31

**Authors:** Amith Khandakar, Muhammad E.H. Chowdhury, Rashid Ahmed, Ahmed Dhib, Mohammed Mohammed, Nasser Ahmed M A Al-Emadi, Dave Michelson

**Affiliations:** 1 Electrical Engineering Department, College of Engineering, Qatar University, Doha-2713, Qatar; mchowdhury@qu.edu.qa (M.E.H.C.); ad1404376@student.qu.edu.qa (A.D.); mm1207599@student.qu.edu.qa (M.M.); alemadin@qu.edu.qa (N.A.A.-E.); 2 Industrial and Mechanical Engineering Department, Qatar University, Doha-2713, Qatar; rashid.ahmed@qu.edu.qa; 3 Electrical Engineering Department, University of British Columbia, Vancouver, BC V6T 1Z4, Canada; davem@ece.ubc.ca

**Keywords:** driving behavior, real-time monitoring, driver distraction, mobile application, portable system

## Abstract

There is an utmost requirement for technology to control a driver’s phone while driving, which will prevent the driver from being distracted and thus saving the driver’s and passenger’s lives. Information from recent studies has shown that 70% of the young and aware drivers are used to texting while driving. There are many different technologies used to control mobile phones while driving, including electronic device control, global positioning system (GPS), on-board diagnostics (OBD)-II-based devices, mobile phone applications or apps, etc. These devices acquire the vehicle information such as the car speed and use the information to control the driver’s phone such as preventing them from making or receiving calls at specific speed limits. The information from the devices is interfaced via Bluetooth and can later be used to control mobile phone applications. The main aim of this paper is to propose the design of a portable system for monitoring the use of a mobile phone while driving and for controlling a driver’s mobile phone, if necessary, when the vehicle reaches a specific speed limit (>10 km/h). A paper-based self-reported questionnaire survey was carried out among 600 teenage drivers from different nationalities to see the driving behavior of young drivers in Qatar. Finally, a mobile application was developed to monitor the mobile usage of a driver and an OBD-II module-based portable system was designed to acquire data from the vehicle to identify drivers’ behavior with respect to phone usage, sudden lane changes, and abrupt breaking/sharp speeding. This information was used in a mobile application to control the driver’s mobile usage as well as to report the driving behavior while driving. The application of such a system can significantly improve drivers’ behavior all over the world.

## 1. Introduction

Over the last few years, road accident incidents have seen a tremendous increase mainly associated with driver distraction caused by mobile phone calls. The U.S. federal government has reported [1,2] that more than 30,000 people are killed on U.S. roads every year in crashes related to distracted driving. Despite knowing the risk of using a mobile phone while driving, 70% of drivers use a mobile phone during driving. There have been recent studies showing the effect of popular social networking games, such as Pokémon GO, leading to many accidents, where users play games while driving [3]. The chances of crashing become higher if mobile phones are used because this reduces driving performance. The control of the sidelong and longitudinal position of the car is one of the basic needs during safe driving; however, frequent use of mobile phones while driving results in poorer lane-keeping, slower response time, and more variable speed [4,5]. In addition, the distance between the driver car and the front car, the fact of wandering out of the driving lane, and a reduced awareness of surroundings are some factors that lead to frequent road accidents and mortalities. In order to control the abrupt use of mobiles phones while driving, we need a smart portable system, which can control and monitor the behavior of a driver whenever the distracted driving exceeds a threshold and the risk of road crashing becomes imminent.

The consequences of using a mobile phone during driving are more lethal than communicating with fellow passenger, and research has shown that drivers busy in discussions over the phone have missed highway exits four times more frequently than those talking with passengers. The drivers talking with travelers did not demonstrate any significant differences with the lone drivers in the simulator environment [6]. The use of cell phones for chatting, messaging, playing media, web browsing, gaming, using the global positioning system (GPS), or working other telephone applications or apps is a dangerous act leading to distracted driving and road crashes [6]. This was evident from a report in 2010 from the U.S. National Highway Traffic Safety Administration (NHTSA) stating that 995 drivers died only because of distraction caused by the use of phones. Similarly, another study in March 2011, carried out by a U.S. insurance agency, State Farm Insurance, declared that 19% of the drivers involved in road accidents were busy talking on a cell phone while driving [7].

The tendency of mobile phone use during driving is more frequent in youth, with one of the studies reporting that more than 90% of college students were involved in initiating, reading, or replying to messages while driving. Messaging while driving, is considered the most harmful among all types of distraction using a mobile phone while driving, and it has been reported that there is a six-fold increase in distraction-related crashes due to text messaging [7]. Mobile phone texting, using MP3s, and other distractions may hinder the capacity of young drivers to control the vehicle and their ability to anticipate and manage hazards [8]. However, collision avoidance systems, electronic stability control, vehicle tracking systems, and intelligent speed adaption may help to reduce the problem even though technology alone cannot make the young driver safe.

A study [9] was conducted that was based on a self-reported questionnaire survey carried out among 242 young drivers in Riyadh, Saudi Arabia to obtain detailed insights into traffic violations committed by young Saudi drivers. The study showed that excessive speeding, which is mainly caused due to running late or testing a car’s performance, is the leading cause of traffic accidents and traffic violations. Moreover, driving very close to the front car, which inhibits the driver to stop in an emergency, is another significant factor leading to traffic accidents. A study was carried out at the American University of Beirut (AUB), Lebanon and at George Washington University (GWU), United States of America to investigate the differences in red-light violations and driving behavior of drivers in those two countries. It has been reported that AUB students engage more in dangerous driving behavior than GWU students do, whereas GWU students are prone to violate traffic rules and red-light signals in the simulator [10]. A study was carried out on 83 new license holder young drivers in private cars, where the system was acquiring driving performance including secondary task engagement and driving environment logging. This study showed that teenage drivers are frequently engaged in secondary tasks and tend to regulate themselves poorly based on the surrounding environmental conditions. Moreover, the teenagers are greatly influenced by peers with respect to engaging in secondary tasks [11]. A system was demonstrated in the study [12], which merged the driver’s background data and driving data to assess the good/bad driving pattern. The score for driving performance managed through the in-vehicle smart system, which provided feedback to the drivers to improve their driving, was found to be useful for taxi companies. There have also been studies where the authors have tried to detect driver distraction using semi-supervised machine learning without developing a prototype [13].

Various technologies and smartphone applications have been developed in recent years in order to limit the frequent use of mobile phones during driving. One of these is Google Glass, but it is still not safe to use [14]. In this same vein, a three-axis accelerometer of an Android-based smartphone was built up with multiple sensors to improve a driver’s awareness to maximize safety [15]. A hardware device that can detect mobile phone use while driving and later block mobile communications could be a very fruitful option for monitoring and controlling road accidents. For example, radio frequency identification (RFID) technology could be used to record the data and send the vehicle’s plate number to a control center when the driver uses the mobile phone and a radio frequency (RF) blocker can be used to block the mobile phone. However, the regulatory commission of some countries does not allow an RF blocker or jammer to be implemented in the car. The use of smartphone accelerometer sensors is another important technique to monitor vehicle status that involves the application of a principal component analysis (PCA) algorithm with time, frequency as well as power spectral density features of the sensor data to predict the vehicle status. This mobile sensor proved beneficial in identifying driving behavior using mobile phone applications [16]. However, this requires a high-performance computational capability in the smartphone application, or the application must be implemented in the cloud. The low-speed following mode (LSM) uses millimeter wave radar to identify the speeding up, deceleration, and stopping of the front car to appraise the distance from the front car; in the interim, the driver likewise controls the brake and the fuel systems to keep up the vehicle distance within the safety range. When the front car encounters a strange condition, the system produces an alert sound to warn the driver [17]. However, this assistive technology is implemented in some expensive cars and is not easy to implement in all available vehicles. The Lane Keeping Assist is a useful system to monitor the passing or separating lane by using a camera fixed to the front of the vehicle. The system produces a cautioning signal when the driver crosses or enters the opposite side of the passing line without using the correct light direction indicator [17]. However, this camera-assisted system is prone to making mistakes during rough weather conditions and bad road conditions, and the image processing requires a powerful computer to be installed on board. In a very recent work [18], a content analysis was conducted on 29 English smartphone applications to identify the stopping, preventing, or reducing phone use behavior while driving, detected by the applications. The functionality of these applications was determined based on application–mobile phone interaction, application–driver interaction, and application–context interaction. Most of these applications focused on blocking specific phone functions; however, the applications did not focus on simplifying phone tasks while driving and none of them was designed to study driving behavior. Another recent work by Delgado et al. [19] showed that the strongest perceived benefit of cellphone blocking apps was decreasing distraction (86%). The predominant reason among young drivers for not wanting to use this technology was not wanting parents to monitor their behavior (60%). This work shows the importance of developing driver-friendly applications while controlling a driver’s mobile usage. The systems that have been reported so far in the literature did not present a feasible solution that could acquire the driving behavior from the car and use it to control the mobile phone automatically.

In this work, we have proposed a hybrid (hardware and software combined) solution to monitor driving behavior and keep track of a driver’s mobile phone usage, and to control the mobile phone call when the car speed reaches a certain threshold. The on-board diagnostics (OBD)-II port of the vehicle was used to get the vehicle’s real-time data. It was used to obtain the car speed very accurately, the accelerometer (ACM) sensors were used to identify some aspects of the driving behavior, and a mobile application written in the Android platform was used to monitor, log, and report driving behavior and control mobile phone calls. We decided on several driving behaviors to be studied and monitored in this work, based on an anonymous self-reported survey that was conducted with 600 male and female teenager drivers belonging to different nationalities. The survey was done in order to determine the pattern and frequency of mobile phone use while driving, and to find out about driving behavior, distracted driving due to mobile phones, and drivers’ level of recommendations regarding the use of technologies to assist drivers or control mobile phones in Qatar. 

## 2. Experiment Details and Methods

In this section, we provide the details of the pilot study conducted to gather self-reported information regarding teenager driving practice in Qatar. Although this survey was conducted for Qatar, this result should reflect the driving practices of Middle East and North African (MENA) countries very closely. This section also provides a detailed description of the hardware and software design of the prototype system used to monitor and control driving behavior. 

### 2.1. Pilot Study

**Figure A1:**
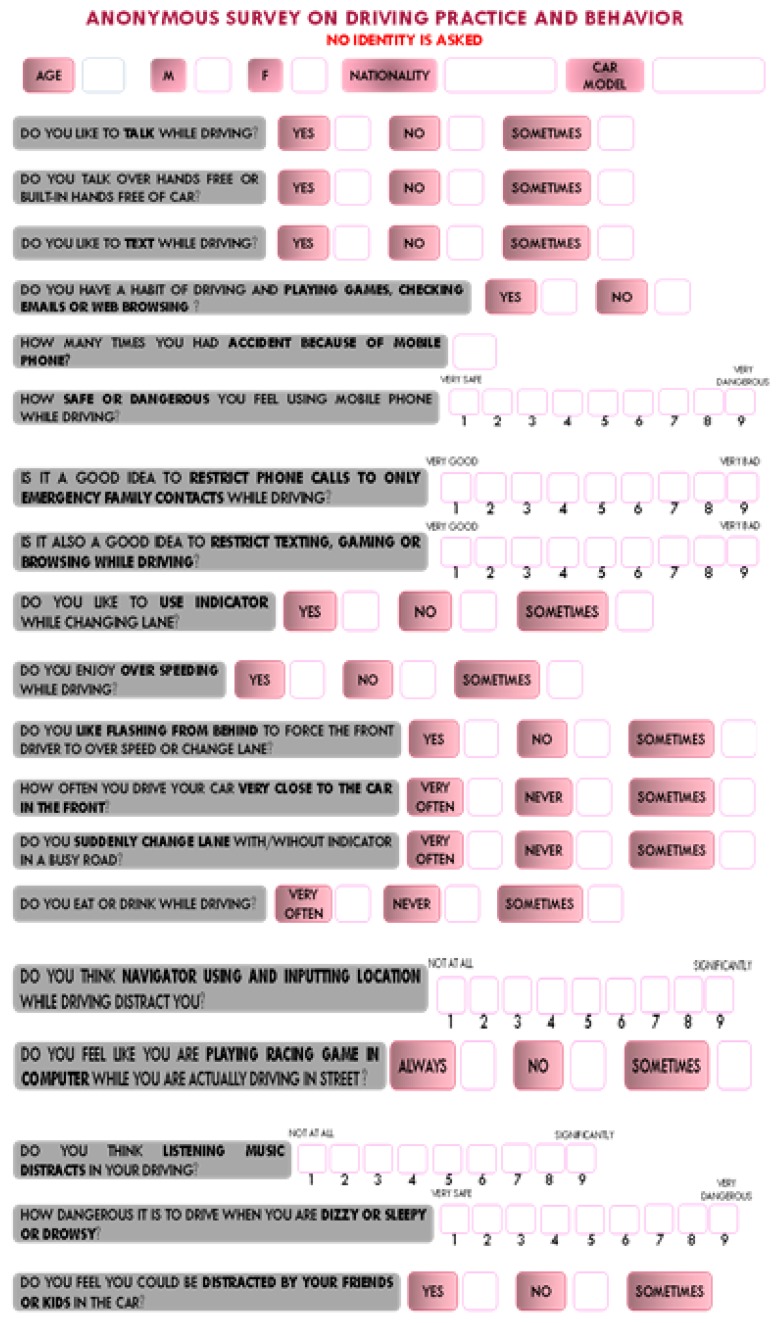
Survey questionnaire.

A self-reported paper-based pilot study was carried out in order to assess the driving behavior and perception of using a mobile phone while driving in Qatar. Both male and female subjects aged 18 to 26 years belonging to different nationalities were selected and data were collected from a population of 600 subjects. An approval was obtained from the Qatar University Human Research Ethics Committee prior to the start of the study. The participants were selected from different undergraduate classes from the universities of Qatar and were given a paper-based questionnaire to choose answers anonymously [13,14,15,16,17,20]. The survey was conducted in both an Arabic and an English version based on the user’s choice (the English version of the survey questionnaire is shown in the Appendix Figure A1). Instructions were given to the participants that the study was to determine the perception of teenage drivers in Qatar about the use of mobile phones while driving. The questionnaire was prepared carefully so that there was no repetitive questions. Additionally, participants were asked to fill in the questionnaire honestly by circling or ticking the number that best suited their opinion after going through it carefully. Extreme care was taken to maintain the anonymity of the study and the confidentiality of the responses by preventing the identification of data obtained from the participants. Furthermore, the identification of participants was prevented by analyzing and reporting the data in a cumulative manner. Moreover, the survey was carried out to address the question of whether the driving behavior played any role based on gender, age range, and nationality or the car model being driven (expensive or inexpensive). 

### 2.2. Design of Experiments

**Figure 1:**
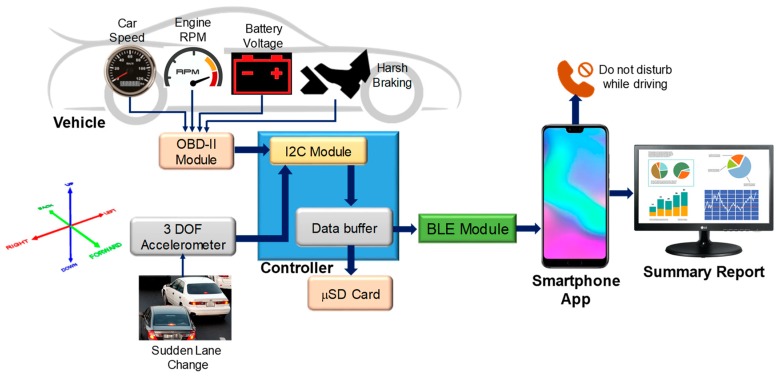
Complete system block diagram.

A complete block diagram of the prototype is shown in Figure 1. Any vehicle manufactured after 1996 is equipped with an on-board diagnostics (OBD) II system, which allows access to the vehicle’s real-time information from its electrical control unit (ECU). The vehicle information from the OBD-II module was sent to a controller unit (microcontroller) and stored in the secure data (SD) card and transmitted via Bluetooth to the mobile phone. The hardware module was powered by the OBD-II module, which took power from the OBD-II port. Therefore, the system only ran while the vehicle was running and it did not drain the vehicle’s battery. There was a three-degree-of-freedom (DOF) accelerometer module interfaced with the controller to keep track of the acceleration in the x-, y-, and z-directions. In the mobile phone, an in-house developed smartphone application made decisions based on the information received from the controller. The mobile application was designed to make decisions based on certain set thresholds, which were determined by detailed experiments on different subjects and will be discussed in the next section. 

#### 2.2.1. Hardware Modules

Various hardware modules are discussed in detail below.

**Figure 2:**
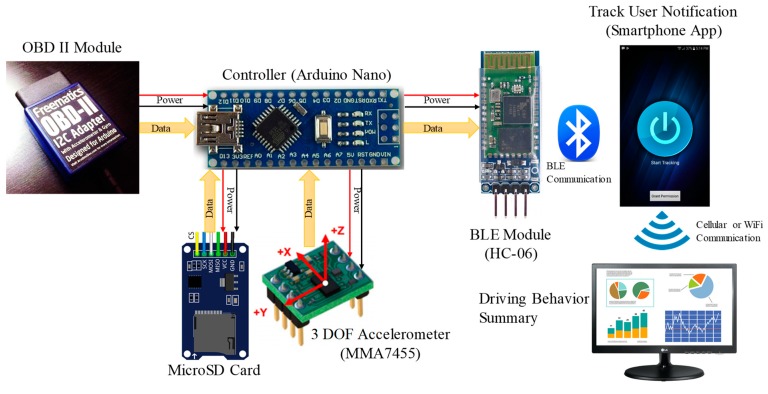
Connection diagram for different modules.

**Figure 3:**
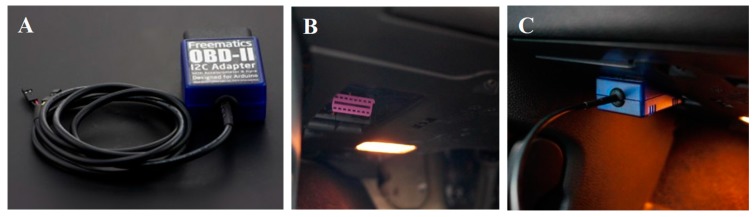
(**A**) On-board diagnostics (OBD)-II module, (**B**) OBD-II port, and (**C**) OBD-II module connected to the car’s OBD-II port.

OBD-II Module: The OBD-II adapter as displayed in Figure 2 was plugged into the OBD port of the vehicle to access various data from the car (car speed, engine rpm, battery voltage, etc.). The data were merged to measure the frequency of sudden breaking-like driving behavior. The connection of the OBD-II module to the OBD port is shown in Figure 3. 

Controller Module: The OBD-II module was interfaced to an Arduino Nano microcontroller to gather the information from it. This information was packaged with 3-DOF accelerometer data and sent to the mobile phone via Bluetooth. The microcontroller and the modules were powered by the car battery through the OBD-II module. The inter-integrated circuit (I^2^C) interface was used for connecting the OBD-II and 3-DOF modules to the microcontroller, whereas the serial peripheral interface (SPI) was used for connecting the Micro SD card module to the microcontroller. 

**Figure 4:**
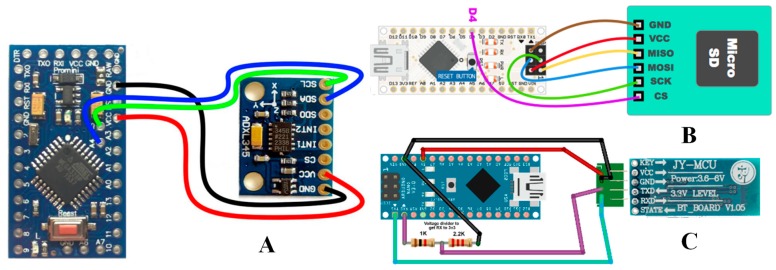
Arduino Nano interfacing wiring with (**A**) MMA7455, (**B**) Micro SD card module, and (**C**) Bluetooth Low Energy (BLE) module.

3-DOF Accelerometer Module: A 3-DOF accelerometer module (MMA7455) was used to collect x- (forward-backward), y- (left-right) and *z*-axis (up-down) acceleration of the vehicle. This was used to identify normal left-right turning, sudden right-left turning, and U-turn. The MMA7455 module was connected with the Arduino Nano using the I^2^C interface (Figure 4A). 

Micro SD Card Module: The information received from the OBD-II and 3-DOF modules was sent to the mobile phone via Bluetooth and stored in a Micro SD card as a backup. This was to ensure that if the connection with the mobile phone and the controller disconnected for some reason, the controller would not lose any data. As soon as the connection was established, the controller updated the mobile application logger to log the information. The details of the connection between the microcontroller and the Micro SD module are shown in Figure 4B. 

Bluetooth Low Energy (BLE) Module: The controller was used to gather useful data (x-, y-, and z-acceleration from the accelerometer and car speed and engine revolution per minute (rpm) from the OBD-II module) continuously every 50 ms with a sampling frequency at 20 Hz, which was sent continuously to the mobile phone. An HC-06 Bluetooth module was used for wireless communication between the controller and the mobile phone. The HC-06 was initially paired with the mobile phone. The microcontroller received the data and combined them in packets and then sent the data to the mobile phone using the HC-06 Bluetooth module. The connection diagram of the microcontroller and the Bluetooth module is shown in Figure 4C. 

#### 2.2.2. Android Mobile Application: Track User Notification

**Figure 5:**
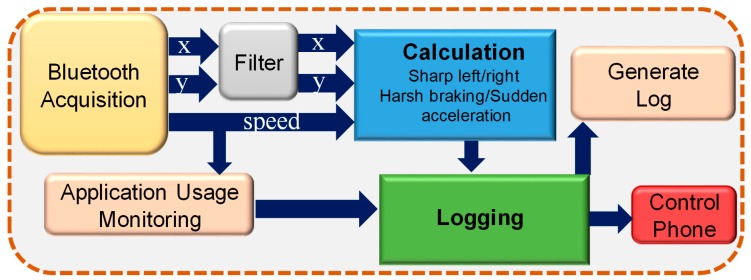
Block diagram to show the stages of the application’s operation.

A tracking application in the Android platform was developed to read the data from the hardware wirelessly, log the data locally for 24 h, and identify the driver’s behavior based on the logged data and pre-set threshold. The application was designed to monitor car speed and control the driver’s mobile phone by restricting receiving or generating phone calls and monitoring behavior while driving if the car speed went above 10 km/h. The application logged only the driver’s other mobile usage information like texting, browsing, playing, etc. At midnight, the application automatically sent the obtained information about car speed, sudden lane change behavior, call blocking, and the driver’s mobile usage to a pre-specified email address. Although the driver was not allowed to generate or receive calls if the speed was above 10 km/h, they could receive a call if the speed dropped below that threshold. Different stages of the mobile application’s workflow are shown in Figure 5.

**Figure 6:**
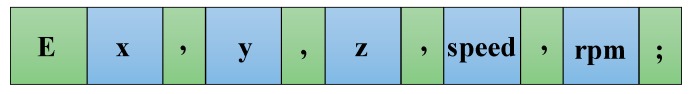
Data packet template.

BLE Acquisition: The Arduino Nano in the hardware module received the data and combined them in a packet and sent the data to the mobile phone using the HC-06 Bluetooth module. The data packet template used for the BLE communication is shown in Figure 6. In the packet template, “E” is a header, “,” is a data separator, and “;” is a data terminator. As shown in Figure 5, there is a module for Bluetooth data reception, and a call constant function was developed to check whether Bluetooth was on or not. 

**Figure 7:**
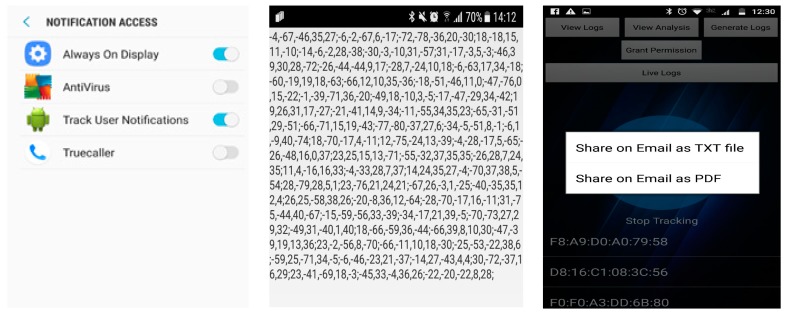
Screen shots of some of the mobile application’s features.

Grant Permission: In order to respect the user’s privacy, the application first asked the user for permission to access and track by enabling the button during the installation process (Figure 7 (left)). If the permission from the user was enabled and Bluetooth was enabled, the application continued. If it found either one disabled, it exited instead of continuing the process.

Automatic Tracking: The app was designed so that it started automatically to track the driver’s mobile phone if the car speed increased above 10 km/h. 

Data Logging and Reporting: The user could see the data acquired from the Bluetooth module using the user interface (Figure 7 (center)). The user could also share the day’s log by email as a text file or as a portable document format (PDF) file, as shown in Figure 7 (right). However, the log was reported automatically to a pre-specified email address at midnight.

**Figure 8:**
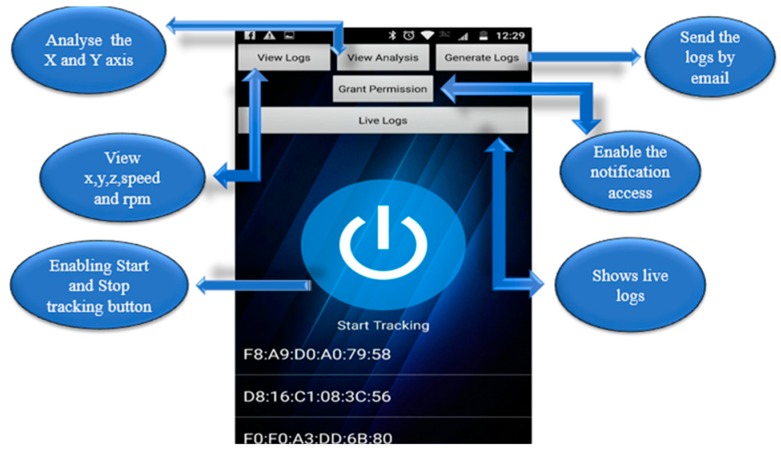
User interface of the Track User Notification mobile application.

The application home interface had five features (and one button for manually starting the tracking), as shown in Figure 8. 

A set of tests were designed to study the performance of the prototype system.

#### 2.2.3. Study 1: Hardware Module Evaluation using Emulator

**Figure 9:**
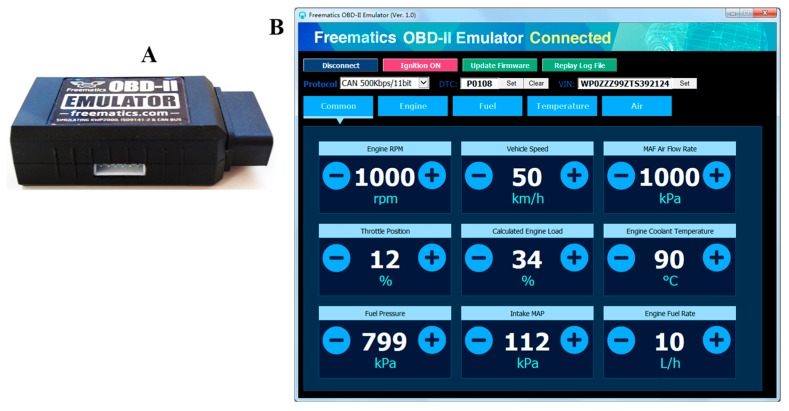
Freematics OBD-II Emulator MK2 (**A**) and Graphical User Interface (GUI) (**B**).

The prototype hardware was initially tested using an emulator before using it on a real car. Freematics OBD-II Emulator MK2 (Freematics, Wahroonga, New South Wales, Australia) (Figure 9A) is an OBD-II emulator with controlled area network (CAN) bus simulation that provides a 16-pin female OBD-II port identical to that of a real car. This device is very useful to get the car’s OBD-II facility on the desk to simulate the real car behavior.

An open-source GUI software is available to see the car parameters on screen and vary them to check the OBD-II device’s performance on the desk. A series of tests were carried out using the emulator to check the performance of the complete system before testing it on a real-car environment. 

The performance of the Bluetooth communication between the vehicle and the controller was evaluated by displaying the received packet from the emulator to the controller (interfaced to a personal computer over a USB interface), and the performance between the controller and the mobile phone was evaluated by analyzing the received packet in the mobile phone, comparing it with the packets sent from the controller. 

#### 2.2.4. Study 2: Hardware Module Evaluation in Vehicle Environment

Experiments were conducted to evaluate the performance of the hardware prototype in reliably acquiring the vehicle information and driving behavior in the real-car environment. The speed of the vehicle reported by the OBD-II module was logged and the speed displayed in the dashboard was recorded synchronously and an off-line comparison was done. 

#### 2.2.5. Study 3: Evaluation of Driving Behavior 

This study was designed to obtain the thresholds for lane change and sudden acceleration/braking behavior of the driver using the data acquired from the OBD-II module and the accelerometer. These thresholds were used to make decisions about driver behavior, such as sharp left, sharp right, sudden brake, or sharp acceleration. We asked ten teenage drivers to perform a series of driving experiments in the pattern listed in Table 1. The OBD-II and accelerometer data were recorded for the various user trials and the threshold for each case was calculated.

#### 2.2.6. Study 4: Evaluation of the Phone Control, Data Logging, and Reporting 

Finally, the application was tested to check if it was working with all the log viewing features and could properly report all the activities through email or not. The initial testing of the application was accomplished by monitoring and tracking all the tasks performed by the driver while data were sent from the controller. We evaluated whether the call controlling feature was activated at 10 km/h or not and whether the user’s activity information (incoming, outgoing, duration of call, etc.), all push notifications (SMS, social media apps, third party messaging apps, that is, any conversation, incoming and outgoing, duration of that session of activity) was correctly logged or not. In addition, we checked whether the app could generate a summary of call activities and notifications and send it automatically by email or not. 

## 3. Analysis

In this work, survey data analysis was accomplished in Microsoft Excel 2016 and vehicle data were initially analyzed in MATLAB 2018 and later (after the development of the mobile application) done in the mobile phone. Initial development and testing of the smartphone application were carried out on a Samsung Note 8 mobile phone, which is powered by an Exynos 8895 Octa-core processor, along with 6 GB of RAM and 64 GB internal memory. The operating system installed on the phone was Android 7.1.1 (Nougat) and enabled with Bluetooth Low Energy (BLE) 5.0. However, the smartphone application was tested in several lower-end smartphones in the testing phase. 

Survey Analysis: Detailed chi square statistics [21] were performed on two major questions, namely, (i) Is it also a good idea to restrict texting, gaming, or browsing while driving and (ii) Is it a good idea to restrict phone calls to only emergency family contacts while driving?, to check the accuracy of the predictions made by the authors based on the literature review. The first question had the assumption that all participants would state that it was a very good idea. For the second question, the authors assumed that all participants would state that it was not safe. 

**Figure 10:**
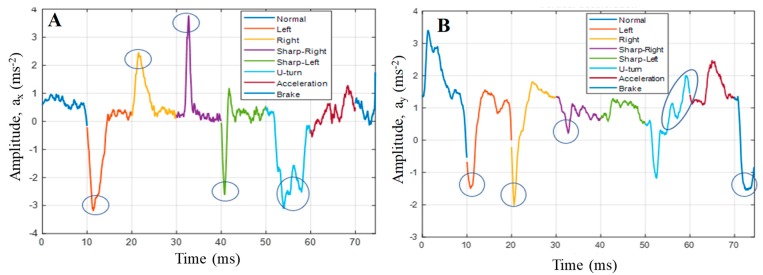
Average x and y data to show (**A**) left/right movements and (**B**) acceleration/braking of the vehicle.

Preliminary Analysis in MATLAB: Initial accelerometer results were smoothened by the moving average filter in MATLAB and averaged over trials and subjects to calculate the mean of the x-, y-, and *z*-axis data (Figure 10). It was observed that, for this work, *z*-axis data were not useful and therefore not used for further processing. Moreover, engine rpm had an offset value when the vehicle was started and changed from that reference and the variation reflected in the engine rpm was also reflected in the speed and therefore rpm was not used for calculating behavior. The accelerometer and OBD-II module data were analyzed to see the trend of driver behavior in relation to the nature of the x and *y*-axis and speed data. 

Data Analysis in Mobile Phone: The mobile application used here to collect the vehicle data from the OBD-II device and to store them temporarily in the mobile memory until they were processed to make a decision (i.e., the x, y, and speed data) were buffered in the mobile memory. The buffering duration was kept to 10 s to get enough data to observe the changes while not cluttering the memory of the mobile phone. The algorithm of real-time peak detection is very robust because it constructs a separate moving mean and deviation for the buffered data, such that signals do not corrupt the threshold. Future signals are therefore identified with approximately the same accuracy, regardless of the number of previous signals. The algorithm takes three inputs: lag represents the lag of the moving window, threshold represents the z-score at which the algorithm generates peak, and influence represents the influence (between 0 and 1) of new signals on the mean and standard deviation. For example, a lag of 5 (moving window) will use the last five observations to smooth the data. A threshold of 3.5 (estimated from MATLAB study) will signal if a datapoint is 3.5 standard deviations away from the moving mean. In addition, an influence of 0.5 gives signals half of the influence that normal datapoints have. Likewise, an influence of 0 ignores signals completely for recalculating the new threshold. An influence of 0 is therefore the most robust option; putting the influence option at 1 is least robust.

**Figure 11:**
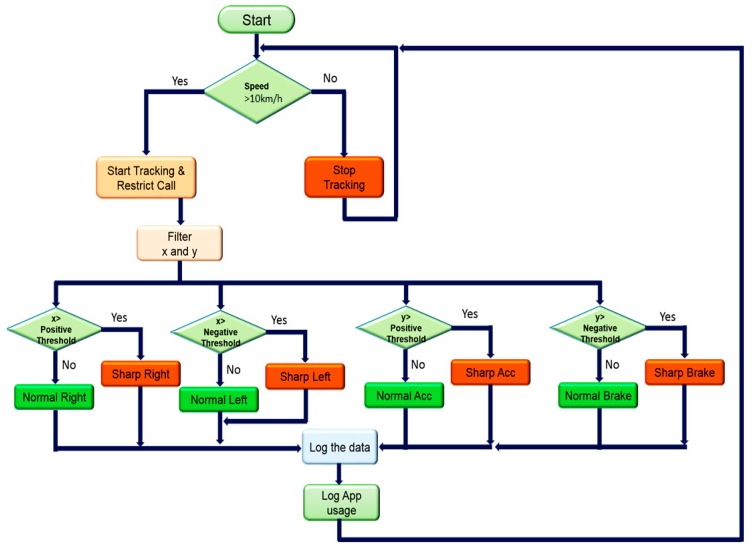
Flowchart of the mobile application’s decision stages.

In the mobile application, there were three subclasses: two for x and y data analysis and the third for the speed data analysis. The subclasses for the x and y data analysis helped to identify the peak of the x and y movements of the vehicle which, in turn, helped to identify normal and abnormal behaviors of the driver, whereas the other subclass sent responses based only on speed data. The filtered speed data were sent to another class, which made a decision based on speed, that is, the vehicle was either in driving mode or stopped, and sent a callback to this class on the current status. Based on the status of the vehicle, the control function started tracking or stopped tracking and the calling function was also deactivated or activated. Figure 11 shows the detailed stages of how the mobile application was designed to log normal or abnormal behavior. 

## 4. Results and Discussion 

The outcomes of the different experiments are summarized in this section.

### 4.1. Summary of Pilot Study

**Figure 12:**
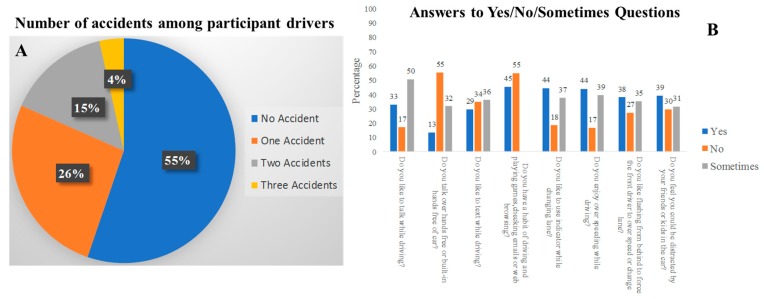
(**A**) Statistics of number of accidents and (**B**) results of some yes/no/sometimes questions among the survey participants.

Some important findings from the survey are presented below, and it is interesting to see that 55% of the survey participants had never had any accidents (from Figure 12A) and thus can be considered as careful drivers. Their answers to the survey questions provided motivation for this work and suggestions to improve the cases of accidents due to mobile phone use. Almost half of the teenage drivers who had received their license within three to six years experienced accidents due to their use of a mobile phone. It can be seen from the self-reported survey percentages shown in the bar chart (Figure 12B) that the drivers were occupied with various activities using the mobile phone during driving. This is one of the primary causes of accidents where the user gets distracted. From Figure 12B, it is evident that 83% of the teenagers like to talk, while 66% of them like to text to some extent while driving. 

**Figure 13:**
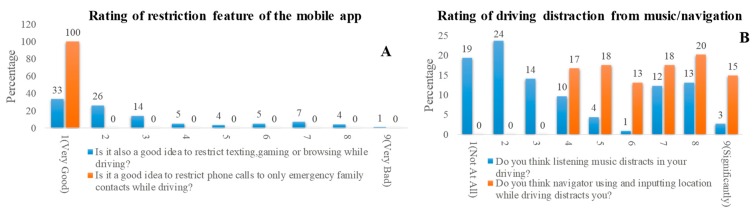
(**A**) Statistics for the questions on rating of restriction feature of the mobile app and (**B**) using mobile phone.

As shown in Figure 13A, the histogram shows that 100% of respondents believe that restricting phone calls except for emergency family calls while driving is a very good idea. However, the restriction of texting, gaming, or browsing was not considered a good idea by all the teenage drivers, although those activities cause more distraction than calling. This clearly highlights the driving behavior of the teenagers. In the same manner, Figure 13B shows that the young drivers mostly do not agree that music can distract the driver, although this point is evident from different research studies conducted in other regions. However, the majority of them agree that using and inputting data to the navigator while driving distract the driver.

**Figure 14:**
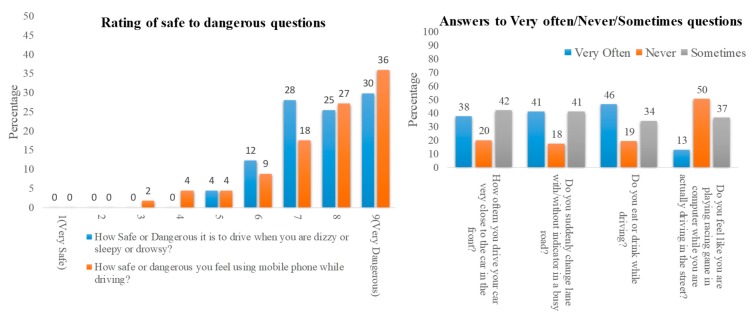
(**A**) Statistics for the rating of safe to dangerous questions and (**B**) results for some very often/never/sometimes questions among the survey participants.

From Figure 14A, it was found that the majority of the survey participants believe that it is not safe to drive while using a mobile phone or when sleepy/drowsy. It can be observed from the self-reported survey percentages shown in the bar chart in Figure 14B that the drivers are occupied with various dangerous activities during driving.

The summary of the chi-squared distribution analysis [19] done on the data is shown in Table 2, where it is observed that such a system is needed for the welfare of drivers and that it is important to raise awareness among teenage drivers in Qatar. 

Comparing the predictions with the actual response and then calculating the chi-squared value of the difference between them and the tabular chi-squared value (for the sample data) for the first question, it was found that the prediction should not be rejected (as the calculated chi-squared value was less than the tabular chi-squared value). However, comparing the predictions with the actual response for question two, it was found that the prediction had to be rejected (as the calculated chi-squared value was more than the tabular chi-squared value).

### 4.2. Performance Evaluation of the Prototype 

#### 4.2.1. Studies 1 and 2

**Figure 15:**
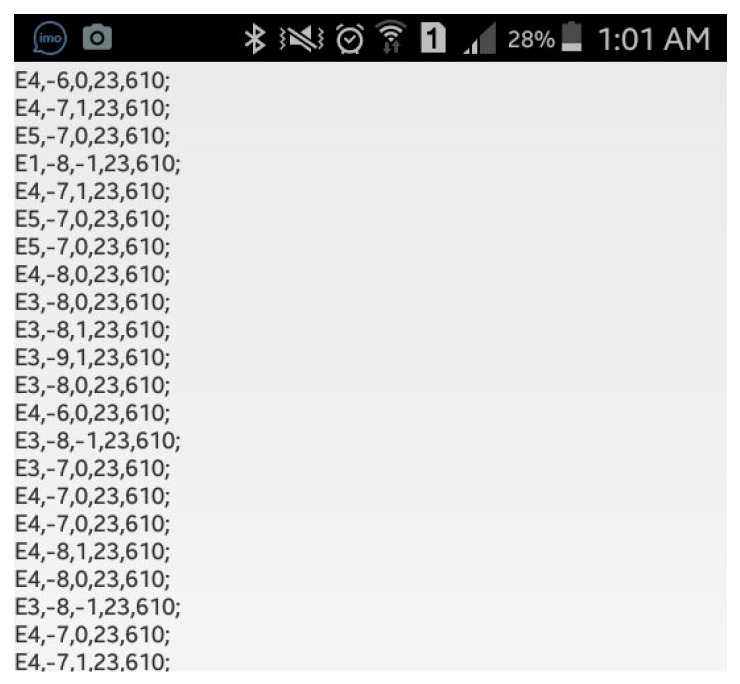
Sample data packets received in the mobile application from real-car testing.

Initial results from the testing of the prototype module using the car emulator along with the OBD-II module showed that the data packets received in the PC match the data sent from the emulator without missing any packets. Moreover, the data sent from the controller to the mobile application were also tested to evaluate their reliability. Figure 15 illustrates the real-car data (x, y, z, speed, rpm) received reliably on the mobile application using Bluetooth communication. 

#### 4.2.2. Study 3: Evaluation of the Driving Behavior

After a series of tests with teenage drivers using the hardware, it was found that there was a specific threshold of the x data, y data, and x, y data combined from the accelerometer, which can help in identifying the driving behavior. The application identified the sudden changes of the x data to classify any changes above the pre-specified threshold to detect “Left”, “Right”, “Sharp Right”, “Sharp Left”, and “U-Turn”. However, only two classes, namely, “Sharp Right” and “Sharp Left”, were logged. Moreover, filtered *y*-axis data helped to classify normal and abnormal acceleration and braking action. If the positive change exceeded the positive threshold, it was classified as “Sudden Acceleration”, and if there was any negative change which occurred below the negative threshold, it was classified as “Sudden Braking” and was logged.

**Figure 16:**
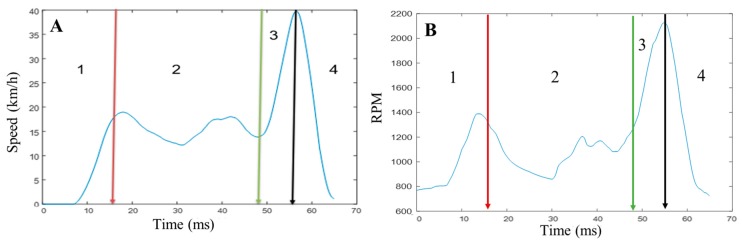
(**A**) Average speed and (**B**) engine rpm for speed monitoring.

According to Figure 16, the speed increased over period number 1, whereas for period number 2 there was no significant change in speed and it remained almost stable. However, over period 3 the speed increased rapidly and reached its maximum value (40 km/h). Finally, over period 4 it decreased sharply to its minimum value (zero km/h). Almost similar information was reflected from the rpm data. This can be used to track excessive speeding behavior if the application is pre-loaded with the speed limit of the particular street along with a global positioning system (GPS). 

#### 4.2.3. Study 4: Evaluation of the Phone Control, Data Logging, and Reporting 

**Figure 17:**
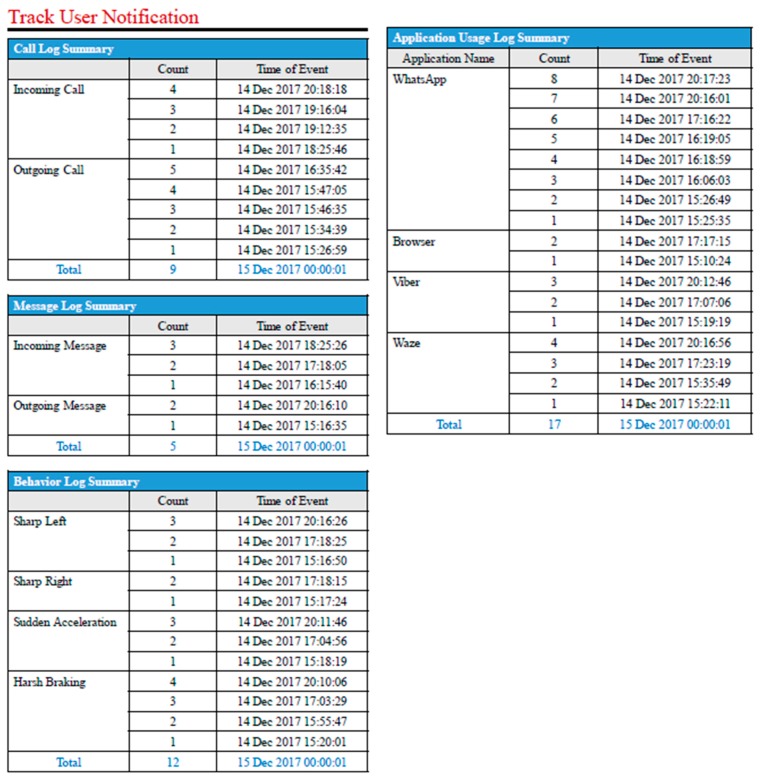
Screenshot of report generated at the end of the day.

Figure 17 shows the Call, Message, Behavior and Application Usage Log Summary while driving for several trials in a day and logged in the application (Tracking User Notification). For example, the Call Log Summary notification shows the call type, Count, and Time of Event. Moreover, the Message Log Summary notification shows the message type (Incoming and Outgoing), the number of total messages, and the time. 

The most important implemented feature of the “Track User Application” mobile app is that it checks the speed of the driver’s vehicle and, if the car speed goes above 10 km/h, it blocks the mobile phone’s call feature. It blocks both the incoming and outgoing mobile calls. This feature was tested on the desk using the OBD-II emulator and also during real driving scenarios. It was observed that in all scenarios, this call blocking feature worked with 100% accuracy. The Call Log Summary in Figure 17 shows that the user attempted to receive and make calls; however, the person was not successful in making/receiving calls because of the enabled blocking feature. However, the mobile application created logs of the incoming/outgoing call attempts in the report. In the report, where the incoming and outgoing call timings were shown, the user was not able to make/receive calls because of the call blocking feature. The Behavior Log Summary shows a summary of some of the driving behavior for that particular day which included sudden left turn, sudden right turn, sudden acceleration, and harsh braking as well as the date and time of the action. This can clearly reflect driving behavior of a particular driver and can easily be modified to monitor accidents as well. It is evident that thresholds help to populate driving behavior from the real-time data. The output from the mobile application was sent to a pre-specified user via email. Moreover, it shows the timing and use of different applications by the mobile user and, more importantly, none of the user privacy data were shared.

## 5. Conclusions

This work proposed a portable solution to gather vehicle details from the ECU of a vehicle through the OBD-II port and to send data to a mobile phone along with an on-board 3-DOF accelerometer to detect driving behavior. The results from the test subjects show that this can be potentially used to identify drivers’ abnormal behaviors. This abnormal driving behavior along with continuous speed monitoring could be used in the mobile application to make decisions on controlling mobile phone activity. The literature reviews and the surveys conducted with a group of teenage drivers in Qatar support the need for such a portable solution. Most of the applications available on the market share the contents of the text, email, or social media message while logging the notifications, whereas our portable solution along with the tracking application does not share any private data in the report, which improves the security of the user. Therefore, the proposed system can be used as a robust system for monitoring the behavior of drivers and controlling them to avoid emergencies. It is clear from the literature and the results of the conducted survey in this work that teenage drivers are willing to stop using their mobile phone while driving. However, incoming messages and calls encourage the user to respond in most of the scenarios and this was observed in the behavior report from the proposed system. The authors therefore consider that significant media awareness through different forms of social media activities along with government and law enforcement involvement (e.g., seat-belt usage enforcement) should be put in place to avoid the life-threatening use of mobile phones while driving. In the future, we plan to add collision detection based on the accelerometer data and the enforcement of Bluetooth for the mobile phone turning-on feature. The system can be modified to allow the user to drive only when Bluetooth is on, thereby enabling the tracking. Further investigation is needed for real-time monitoring of driving behavior, where the system is deployed in several vehicles and monitored for a longer duration to truly benefit from this research.

## Figures and Tables

**Table 1:** Driving pattern for data collection to calculate the thresholds.

Duration	Actions
0 s–10 s	Normal
10 s–20 s	Turn left
20 s–30 s	Turn right
30 s–40 s	Sudden change lane toward right
40 s–50 s	Sudden change lane toward left
50 s–60 s	U-turn(leftward)
60 s–70 s	Sudden acceleration
70 s	Harsh brake

**Table 2:** Chi-Squared distribution results for two major survey questions.

Question	Prediction	Reject or Accept Depending on Chi-Squared Distribution
Is it a good idea to restrict phone calls to only emergency family contacts while driving?	Almost all of them should say that it is a very good idea	Accept, which is a good motivation to the development of the prototype
How safe or dangerous do you feel using mobile phone while driving?	Almost all of them should say that it is very dangerous	Reject which is a good motivation to increase awareness about it among them.

## References

[1] Shabeer H.A., Banu R.W., Zubar H.A. (2012). Technology to prevent mobile phone accidents. Int. J. Enterp. Netw. Manag..

[2] Guo F., Klauer S.G., Fang Y., Hankey J.M., Antin J.F., Perez M.A., Lee S.E., Dingus T.A. (2017). The effects of age on crash risk associated with driver distraction. Int. J. Epidemiol..

[3] Ayers J.W., Leas E.C., Dredze M., Allem J.-P., Grabowski J.G., Hill L. (2016). Pokémon GO—A new distraction for drivers and pedestrians. JAMA Intern. Med..

[4] Burns P., Parkes A., Burton S., Smith R., Burch D. (2002). How Dangerous Is Driving with a Mobile Phone?: Benchmarking the Impairment to Alcohol.

[5] Oviedo-Trespalacios O., Haque M.M., King M., Washington S. (2016). Understanding the impacts of mobile phone distraction on driving performance: A systematic review. Transp. Res. Part C Emerg. Technol..

[6] Coxon K., Keay L. (2015). Behind the wheel: Community consultation informs adaptation of safe-transport program for older drivers. BMC Res. Notes.

[7] Drews F.A., Pasupathi M., Strayer D.L. (2008). Passenger and cell phone conversations in simulated driving. J. Exp. Psychol. Appl..

[8] Lee J.D. (2007). Technology and teen drivers. J. Saf. Res..

[9] Hassan H.M. (2016). Investigation of the self-reported aberrant driving behavior of young male Saudi drivers: A survey-based study. J. Transp. Saf. Secur..

[10] Danaf M., Hamdar S.H., Abou-Zeid M., Kaysi I. (2018). Comparative assessment of driving behavior at signalized intersections using driving simulators. J. Transp. Saf. Secur..

[11] Gershon P., Zhu C., Klauer S.G., Dingus T., Simons-Morton B. (2017). Teens’ distracted driving behavior: Prevalence and predictors. J. Saf. Res..

[12] Cen J., Wang Z., Wang C., Liu F. A System Design for Driving Behavior Analysis and Assessment. Proceedings of the 2016 IEEE International Conference on Internet of Things (iThings) and IEEE Green Computing and Communications (GreenCom) and IEEE Cyber, Physical and Social Computing (CPSCom) and IEEE Smart Data (SmartData).

[13] Liu T., Yang Y., Huang G.-B., Yeo Y.K., Lin Z. (2016). Driver distraction detection using semi-supervised machine learning. IEEE Trans. Intell. Transp. Syst..

[14] He J., Choi W., McCarley J.S., Chaparro B.S., Wang C. (2015). Texting while driving using Google Glass™: Promising but not distraction-free. Accid. Anal. Prev..

[15] Fazeen M., Gozick B., Dantu R., Bhukhiya M., González M.C. (2012). Safe driving using mobile phones. IEEE Trans. Intell. Transp. Syst..

[16] Lu D.-N., Nguyen D.-N., Nguyen T.-H., Nguyen H.-N. (2017). A Novel Mobile Online Vehicle Status Awareness Method Using Smartphone Sensors. Information Science and Applications.

[17] Lu S.-N., Tseng H.-W., Lee Y.-H., Jan Y.-G., Lee W.-C. (2010). Intelligent safety warning and alert system for car driving. Tamkang J. Sci. Eng..

[18] Oviedo-Trespalacios O., King M., Vaezipour A., Truelove V. (2019). Can our phones keep us safe? A content analysis of smartphone applications to prevent mobile phone distracted driving. Transp. Res. Part F Traffic Psychol. Behav..

[19] Delgado M.K., McDonald C.C., Winston F.K., Halpern S.D., Buttenheim A.M., Setubal C., Huang Y., Saulsgiver K.A., Lee Y.C. (2018). Attitudes on technological, social, and behavioral economic strategies to reduce cellphone use among teens while driving. Traffic Inj. Prev..

[20] Ajzen I. (2002). Constructing a TPB questionnaire: Conceptual and methodological considerations. http://chuang.epage.au.edu.tw/ezfiles/168/1168/attach/20/pta_41176_7688352_57138.pdf.

[21] Lancaster H.O., Seneta E. (2005). Chi-square distribution. Encycl. Biostzatistics.

